# Fatigue in Patients with Multiple Sclerosis: Is It Related to Pro- and Anti-Inflammatory Cytokines?

**DOI:** 10.1155/2015/758314

**Published:** 2015-01-19

**Authors:** Arjan Malekzadeh, Wietske Van de Geer-Peeters, Vincent De Groot, Charlotte Elisabeth Teunissen, Heleen Beckerman, TREFAMS-ACE Study Group

**Affiliations:** ^1^Neurochemistry Laboratory, Department of Clinical Chemistry, VU University Medical Center, Neuroscience Campus Amsterdam, P.O. Box 7057, 1007 MB Amsterdam, Netherlands; ^2^MS Center Amsterdam, P.O. Box 7057, 1007 MB Amsterdam, Netherlands; ^3^Department of Rehabilitation Medicine, VU University Medical Center, P.O. Box 7057, 1007 MB Amsterdam, Netherlands; ^4^EMGO Institute for Health and Care Research, VU University Medical Center, P.O. Box 7057, 1007 MB Amsterdam, Netherlands

## Abstract

*Objective*. To investigate the pathophysiological role of pro- and anti-inflammatory cytokines in primary multiple sclerosis-related fatigue. *Methods*. Fatigued and non-fatigued patients with multiple sclerosis (MS) were recruited and their cytokine profiles compared. Patients with secondary fatigue were excluded. Fatigue was assessed with the self-reported Checklist Individual Strength (CIS20r), subscale fatigue. A CIS20r fatigue cut-off score of 35 was applied to differentiate between non-fatigued (CIS20r fatigue ≤34) and fatigued (CIS20r fatigue ≥35) patients with MS. Blood was collected to determine the serum concentrations of pro-inflammatory cytokines (IL-1*β*, IL-2, IL-6, IL-8, IL-12p70, IL-17, TNF*α*, and IFN-*γ*) and anti-inflammatory cytokines (IL-4, IL-5, IL-10, and IL-13). We controlled for the confounding effect of age, gender, duration of MS, disease severity, type of MS, and use of immunomodulatory drugs. *Results*. Similar cytokine levels were observed between MS patients with (*n* = 21) and without fatigue (*n* = 14). Adjusted multiple regression analyses showed a single significant positive relationship, that of IL-6 with CIS20r fatigue score. The explained variance of the IL-6 model was 21.1%, once adjusted for the confounding effect of age. *Conclusion*. The pro-inflammatory cytokine interleukin-6 (IL-6) may play a role in the pathophysiology of primary fatigue in patients with MS. *Trial Registrations*. ISRCTN69520623, ISRCTN58583714, and ISRCTN82353628.

## 1. Introduction

Multiple sclerosis (MS) is an inflammatory demyelinating and neurodegenerative disease of the central nervous system [1]. The age of onset of MS is usually between the age of 20 and 40, and MS affects females twice as often as males [1]. Prevalence and incidence are greater in higher northern and southern latitudes [1, 2]. The disease course of MS can be divided into three main subtypes, known as relapsing remitting (RR), secondary progressive (SP), and primary progressive (PP) [1, 2].

Fatigue is one of the most prominent and disabling symptoms of MS and is independent of MS subtype, restricting patients' societal participation and performance in daily life at home, at work, and in leisure activities [3, 4]. MS-related fatigue can be subdivided into primary and secondary fatigue [4, 5]. Primary fatigue relates to specific pathophysiological mechanisms that are the direct consequence of the MS disease process, such as demyelination and axonal loss [5], while secondary fatigue can be attributed to symptoms or an accumulating disease burden, including sleep disorders, depression, and the side effects of therapy [5]. It is often difficult to differentiate between primary and secondary fatigue as both can be simultaneously present in a patient and may impact each other [5, 6]. A common example is depression due to the major impact of primary fatigue on a patient's quality of life. The depression can then further worsen fatigue.

A better understanding of the pathophysiological mechanisms of primary fatigue would allow the development of effective treatments. A first step towards this goal requires understanding of the biological correlates of primary MS-related fatigue. Optimal pharmacological treatments for MS-related fatigue are currently unavailable and oral therapies such as Amantadine or Pemoline are usually only partially effective [7]. Limited insight into the pathophysiological mechanisms of primary MS-related fatigue is probably an important contributory factor to the current lack of treatment options. In this study we specifically focus on primary MS-related fatigue by excluding secondary fatigue causes.

Multiple mechanisms have been proposed to play a role in primary MS-related fatigue, including a chronic imbalance in inflammatory factors, [5, 8]. Both the serum levels of pro-inflammatory cytokines and fatigue increase during a relapse [3, 4]. Moreover, fatigue is one of the side effects of immune-modulatory medications such as interferon-*β* [5, 9]. Furthermore, an association has been found between fatigue and levels of inflammatory cytokines in other (auto)immune-related diseases, such as rheumatoid arthritis (RA), human immunodeficiency virus (HIV), and systemic lupus erythematosus (SLE) [8, 10, 11]. Finally, cytokines have been related to sleep disorders including sleep apnea syndrome, narcolepsy, idiopathic hypersomnia, and chronic fatigue syndrome [11, 12].

The aim of this study was to explore a possible relationship between serum concentrations of pro- and anti-inflammatory cytokines and primary MS-related fatigue through the quantification of twelve different serum cytokines by electrochemiluminescence-based multiplex immunoassay.

## 2. Materials and Methods

### 2.1. Design

Fatigued and non-fatigued patients with MS were recruited and their cytokine profiles compared. In addition, pro- and anti-inflammatory cytokine concentrations were related to the level of MS-related fatigue.

### 2.2. Participants

All patients included in the study met the following inclusion criteria: (a) definitive diagnosis of MS, (b) age between 18 and 70 years, (c) being ambulatory, and (d) normal thyroid function (blood levels of thyroid stimulating hormone of 0.3–4.5 mU/L). The exclusion criteria for the study were (a) evident signs of an exacerbation or corticosteroid treatment in the past 3 months, (b) current infections (abnormal leukocytes and C-reactive protein in blood), (c) anaemia (abnormal haemoglobin and haematocrit in blood), (d) depression (Hospital Anxiety and Depression Scale, depression score > 11), (e) primary sleep disorders, (f) severe comorbidity (Cumulative Illness Rating Scale (CIRS) item score of ≥3) [13, 14], (g) current pregnancy or having given birth in the past 3 months, (h) pharmacological treatment for fatigue that was started in the past 3 months (e.g., Amantadine, Modafinil, Ritalin, and Pemoline), (i) non-pharmacological therapies for fatigue that took place in the past 3 months.

Non-fatigued patients, defined as having a score of 34 or less on the Checklist Individual Strength (CIS20r) subscale fatigue, were recruited from the Department of Rehabilitation Medicine of the VU University Medical Center or the Rehabilitation Center Heliomare [15, 16]. Patients with a score of 35 or more on the CIS20r subscale fatigue were recruited from the* Treating Fatigue in MS* research programme (TREFAMS-ACE) [16, 17].

The study protocol was approved by the Medical Ethics Committees of the participating centres, with the Medical Ethics Committee of the VU University Medical Center, Amsterdam, acting as the principal review board. Written informed consent was obtained from each participant prior to entering the study.

### 2.3. Clinical Scores

All patients underwent neurological screening to classify the disease course and to determine the Expanded Disability Status Scale (EDSS) score [18]. Furthermore, the presence and severity of comorbidities were quantified using the CIRS [13, 14]. Data on age, gender, and the use of disease-modifying drugs were collected.

### 2.4. Fatigue

Fatigue was measured with the CIS20r, subscale fatigue [15, 16]. The CIS20r is a multidimensional questionnaire that consists of 20 items, divided into 4 dimensions of fatigue and related behavioural aspects: (a) the subjective experience of fatigue (8 items), (b) reduction in motivation (4 items), (c) reduction of physical activity (3 items), and (d) reduction in concentration (5 items). The CIS20r focuses on fatigue in the past 2 weeks and each item is answered using a 7-point scale. The CIS20r fatigue score is a sum score that can vary between 8 and 56 points, the latter corresponding to the maximum level of fatigue [15, 16].

### 2.5. Blood Draw and Sample Handling

Blood was drawn from the participants between 9:00 and 15:00 and at three centres, using the same study protocol and BD Vacutainer plastic serum tubes (BD, New Jersey, USA). The tubes were processed within 1 hour by centrifugation at 1800 ×g for 10 minutes and subsequently stored in polypropylene tubes (Sarstedt, Germany) at −80°C.

### 2.6. Multiplex Cytokine Assay

Evaluation of multiple cytokines took place at the neurochemistry lab of the VU University Medical Center, using the ultrasensitive human Th1/Th2-10 plex kit (Meso Scale Discovery (MSD), Maryland, USA). This kit allows the detection of IL-1*β*, IL-2, IL-4, IL-5, IL-8, IL-10, IL-12p70, IL-13, TNF-*α*, and IFN-*γ*. The cytokines IL-6 and IL-17 were measured using the single-plex human ultrasensitive IL-6 and the human ultrasensitive IL-17 kits (MSD). The MSD kits were run according to the manufacturer's guidelines. The chemiluminescence signal was measured and the read-out performed on a Sector Imager 6000 (MSD). The cytokine concentrations were determined using Discovery Workbench 3.0.18 software from the instrument and a log-log curve fit model. Overall, the average lower limit of detection in serum for Th1/Th2-10 plex kit was 0.67 pg/mL. The overall coefficient of variation (CV) for samples performed in duplicate was <20%. The lower limits of detection of the IL-6 and IL-17 kits were 0.7 pg/mL and 0.2 pg/mL, respectively, with an average CV of <10% and <13%.

### 2.7. Statistical Analysis

Group differences with regard to demographic and disease-related variables were analysed using the independent *t*-test, Mann-Whitney *U* test, or Chi-square test. Because cytokine concentrations were not normally distributed, the cytokines of the non-fatigued and the fatigued patients were compared for statistical significance using the Mann-Whitney *U* test. Log-transformed cytokine values were used in multiple linear regression analyses, with the CIS20r fatigue score as a continuous outcome measure [19]. Age, gender, duration of MS, disease severity (EDSS), MS subtypes, and the use of immunomodulatory drugs were included in the multiple regression analyses to adjust for confounding when appropriate, that is, when these variables individually changed the univariate (i.e., unadjusted) regression coefficient of each cytokine by more than 10% [20]. All analyses were carried out in SPSS 20.0.

## 3. Results

### 3.1. Participants

Thirty-five patients with MS (24 female, 11 male; mean age 44 ± 9.6 years) were eligible to participate. Twenty-six patients had relapsing remitting MS, four patients had secondary progressive MS, and four patients had primary progressive MS (Table 1). The type of MS was not specified for one patient. The median disease duration was 8.6 years (minimum-maximum 0.2–21.2 years). Eighteen patients had no or mild neurological disability, defined as an EDSS of <3, while sixteen patients had moderate or severe disability defined as an EDSS of ≥3. The EDSS was missing for one patient. Sixteen patients had no comorbidity; that is, the total CIRS score was 0, while 19 patients had one or more non-severe comorbid conditions that require (pharmacological) treatment but have a good prognosis. Seventeen of the patients were receiving disease-modifying immune-modulatory treatment, for example, interferon beta-1a, interferon beta-1b, glatiramer acetate, or natalizumab. Twenty-one participants were severely fatigued (CIS20r fatigue ≥ 35), and 14 patients were classified as non-fatigued (CIS20r fatigue ≤34). Patients in the fatigue group also showed reduced concentration, motivation, and physical activity on the CIS20r (Table 1). The fatigue and non-fatigue groups did not differ significantly in age, gender, duration of MS, type of MS, EDSS, or use of immunomodulatory drugs. Thus, the potentially confounding variables were equally distributed among the fatigued and non-fatigued patients.

### 3.2. Cytokines in MS Patients with and without Primary Fatigue

The serum concentrations of pro-inflammatory (IL-1*β*, IL-2, IL-6, IL-8, IL-12p70, IL-17, TNF*α*, and IFN-*γ*) and anti-inflammatory cytokines (IL-4, IL-5, IL-10, and IL-13) are summarized in Table 1. No differences in the profiles of pro-inflammatory cytokines and anti-inflammatory cytokines were observed between the fatigued and non-fatigued groups.

### 3.3. Regression Analyses of Cytokines with CIS20r Fatigue

Multiple regression analyses of the 12 serum cytokine concentrations (log 10 transformed) showed that pro-inflammatory IL-6 was the only cytokine significantly related to the CIS20r fatigue score (*P* value 0.009) (Table 2). Age was identified as confounder of the relationship between IL-6 and the CIS20r fatigue (10% change of the univariate regression coefficient of IL-6). The explained variance (*R* ^2^) of IL-6 on fatigue, adjusted for age, was 21.1%. None of the potential confounders age, gender, duration of MS, disease severity, type of MS, and immunomodulatory drugs showed an independent and significant regression coefficient with the CIS20r fatigue score.

## 4. Discussion

In this study we investigated the pathophysiological role of pro- and anti-inflammatory cytokines in primary MS-related fatigue. Comparison of the serum cytokine levels of 21 patients with severe fatigue with those of 14 patients with low fatigue scores showed only small, non-significant differences. Moreover, multiple regression analysis showed no association of the cytokines IL-1*β*, IL-2, IL-4, IL-5, IL-8, IL-12p70, IL-17, TNF*α*, IFN-*γ*, IL-10, and IL-13 with primary MS-related fatigue, with the exception of the pro-inflammatory cytokine IL-6. Furthermore, fatigue scores were not associated with the patient characteristics age, gender, disease duration, type of MS, EDSS, or the use of immunomodulatory drugs.

The major strength of this pathophysiological study was that we focussed on primary MS-related fatigue by excluding secondary fatigue. This allowed us to assess primary fatigue-associated factors and thus gain insight into the pathophysiological mechanisms affecting primary fatigue. Furthermore, we succeeded in including fatigued and non-fatigued patients. An additional strength was the comparison of patients with the same disease, rather than using a healthy control group or a group of patients with another chronic disease. Moreover, we assessed a broad range of different pro- and anti-inflammatory cytokines by applying the mesoscale discovery multiplex platform, which has been shown in various studies to allow a very reliable and sensitive multiplex assessment of cytokines in body fluids [21–23]. Finally, the associations of cytokines with primary MS-related fatigue were adjusted for potential bias due to confounding factors.

The limitations of this study should also be considered, the main limitations being the small sample of MS patients and the large number of statistical tests in the same set of patients. Due to our small sample size of 35 participants and the anticipated multicollinearity between cytokines, we decided to run 12 “independent” models, with the same fatigue outcome, and in each model one cytokine as explanatory determinant. A close inspection of the correlation matrix of the cytokines showed high multicollinearity (>0.70) between the anti-inflammatory cytokines. However, most of the correlations of the pro-inflammatory cytokines were low and non-significant. With respect to IL-6, the highest correlation was with the log-transformed IL-1*β*, with a Pearson correlation coefficient of −0.358. This might explain why we found an association with IL-6 alone and not with the other immune measures. We were strengthened in our conclusion by the fact that the association of IL-6 with fatigue was also called to attention by previous work in an open population and in other patient populations as we discuss below. As this was an exploratory study, instead of a confirmatory study with an increasing number of family-wise null hypotheses to test, we have refrained from a Bonferroni-like correction to change the significance level *α* = 0.05 [24].

Furthermore, the cross-sectional design of this study did not allow the pathophysiological causality of the cytokines to be established. Blood was drawn between 9:00 and 15:00 and on average 30 minutes earlier in the fatigued patients compared to the non-fatigued group. Cytokines that are sensitive to a circadian rhythm should preferably be measured more than once a day.

Overall, we believe that we have carried out a valid determination of the (lack of) role of 11 serum cytokines in primary MS-related fatigue, adjusting for 6 potentially relevant confounders. In agreement with the trend of our results, an earlier study found no association between the Fatigue Severity Scale (FSS) and the systemic inflammation markers neopterin, C-reactive protein (CRP), and sICAM-1 in the urine and serum of patients with different MS subtypes, compared to healthy controls [25]. Our results contrast with those of some other studies. In whole blood stimulation experiments involving MS patients with fatigue compared to MS patients without fatigue, Heesen et al. showed an elevated productive capacity for TNF-*α* and IFN-*γ* in the former group [8], whereas IL-10 was not significantly different between the two patient groups [8]. The discrepancy with our results may be due to the fact that we measured serum concentrations, whereas the aforementioned study measured stimulated cytokine production capacity [8]. Others also showed a correlation between TNF-*α* mRNA in serum and fatigue but no correlation of IFN-*γ* mRNA with MS-related fatigue [26]. Differences in capacity to produce cytokines between patients with and without MS-related fatigue could potentially be explained by the epigenetic and translational regulation mechanisms that are also involved in TNF-*α* and IFN-*γ* production [27].

Other methodological differences in the measurements of fatigue and the determination of cytokines hamper a direct comparison of studies with regard to the cytokine concentrations and their association with fatigue. An example is our use of the CIS20r subscale fatigue, in contrast to the use of the FSS in the previously mentioned studies [8, 25, 26]. Although the FSS is a useful and rapid fatigue screening tool, its main use is in quantifying the impact of fatigue on daily functioning rather than ascertaining the perceived level of fatigue [28]. Differences in cytokine detection techniques might also have contributed to differing study results. For instance, Heesen et al. [8] used ELISA (enzyme-linked immunosorbent assay) to analyse cytokines, whereas we used a multiplex kit. Other possible explanations for divergent findings across studies may pertain to the specific characteristics of the study populations.

The pro-inflammatory cytokine IL-6 was the only immunological marker in our study that was significantly related to the severity of primary MS-related fatigue after controlling for confounding. There is considerable evidence that the serum concentration of IL-6, as well as those of other cytokines, is influenced by both age and ethnicity [29, 30]. The confounding effect of age on the association of IL-6 with fatigue observed by us is therefore not specific for MS. The immunological function of IL-6 is complex and not exclusively pro-inflammatory [29, 31]. For instance, local production of IL-6 in the medial hypothalamus and preoptic nucleus activates the hypothalamic-pituitary-adrenocortical (HPA) axis and regulates a variety of central effects such as stress, sleep, and fever [29, 31, 32]. Elevated levels of IL-6 in the presence of psychological stress were also seen in patients with MS [33]. A biological measure of stress was not included in our study. In future studies it is worthwhile to further investigate the cause and effect relationship of stress, IL-6, and MS-related fatigue. There is also some evidence from other studies that IL-6 is associated with fatigue. In a non-medical, population-based cohort study, the Whitehall II study, high levels of IL-6 (≥1.5 pg/mL), together with high levels of plasma C-reactive protein (≥1.0 mg/mL), were predictive of new-onset fatigue in participants (aged 39–63 years) after a mean follow-up of 3.1 years [11]. From these results it was concluded that low-grade systemic inflammation may play an important role in the development of fatigue [11]. Associations between IL-6 and fatigue were also found in other patient populations [10, 34]. However, no significant correlations were found between symptoms of fatigue and IL-6 or C-reactive protein in patients with Parkinson's disease [35].

As recently postulated, different MS subtypes could have different underlying disease mechanisms. It is therefore possible that primary fatigue experienced within these subtypes could also be due to different mechanisms [36]. Although the sample size of the current study was too small to allow subgroup analyses, future studies should consider the assessment of each MS subtype with and without perceived fatigue. Assessing the levels of cytokines in other body fluids, such as cerebrospinal fluid (CSF), could help to further unravel the pathophysiological mechanism of MS-related fatigue. Inflammation is a biological response of the immune system to a number of different stimuli. If serum levels of cytokines reflect general systemic inflammation and CSF levels of cytokines are more closely related to disease-specific processes in the central nervous system of patients with MS, simultaneous testing of multiple cytokines in serum and CSF may shed new light on the possible mechanism of inflammation in MS-related fatigue. In addition, the concentration of IL-6 in serum follows a circadian rhythm [29], suggesting that it might be worthwhile to study the circadian rhythm of IL-6 compared with the circadian rhythm of fatigue in patients with MS. Future large longitudinal studies investigating the causal or etiological role of inflammatory markers in primary MS-related fatigue would be more informative when taking multiple rather than single blood and CSF samples.

## 5. Conclusions

This study showed that the pro-inflammatory cytokine interleukin-6 (IL-6) may play a role in the pathophysiology of primary fatigue in patients with MS.

## Figures and Tables

**Table 1:** Clinical characteristics and cytokine scores of fatigued and non-fatigued multiple sclerosis patients.

	Fatigue (*n* = 21)	No fatigue (*n* = 14)	*P* value
*Patient characteristics *			
Gender			0.77^a^
Male	7	4	
Female	14	10	
Age, mean (sd) (years)	43.3 (10.6)	45.0 (8.2)	0.60^b^
Duration of MS, median (min–max) (years)	8.7 (0.2–20.5)	8.6 (1.0–21.2)	1.00^c^
Type of MS^*^			0.81^a^
Relapsing remitting	15	11	
Primary or secondary progressive	5	3	
EDSS, median (IQ)^*^	2.25 (1.13–3.38)	2.75 (1.38–3.13)	0.99^c^
Immunomodulatory treatment			0.89^aa^
Interferon beta	7	3	
Glatiramer acetate	1	0	
Natalizumab	2	4	
No	11	7	
CIS20r mean (sd)			
Fatigue	46.2 (5.0)	20.6 (7.5)	0.00^b^
Concentration	19.3 (9.9)	16.6 (8.9)	0.40^b^
Motivation	14.1 (5.4)	10.4 (6.2)	0.08^b^
Physical activity	12.0 (5.0)	7.9 (3.7)	0.01^b^
*Pro-inflammatory cytokines *			
Median (min–max) (pg/mL)			
IL-1*β*	0.3 (0.1–1.1)	0.3 (0.1–2.9)	0.65^c^
IL-2	0.2 (0.0–1.1)	0.3 (0.0–1.4)	0.21^c^
IL-6	0.4 (0.2–9.7)	0.3 (0.2–0.9)	0.13^c^
IL-8	10.0 (5.2–33.9)	10.0 (3.3–18.5)	0.70^c^
IL12p70	4.4 (0.9–117.6)	3.4 (0.48–229.0)	0.52^c^
IL-17	0.3 (0.0–1.7)	0.2 (0.0–0.8)	0.28^c^
TNF-*α*	7.3 (4.2–12.5)	6.7 (5.1–11.8)	0.78^c^
IFN-*γ*	0.8 (0.2–75.7)	1.0 (0.4–5.0)	0.70^c^
*Anti-inflammatory cytokines *			
Median (min–max) (pg/mL)			
IL-4	1.2 (0.6–2.6)	1.1 (0.6–3.9)	0.75^c^
IL-5	0.5 (0.2–16.0)	0.8 (0.2–15.6)	0.54^c^
IL-10	2.3 (0.5–42.9)	2.0 (0.9–60.8)	0.75^c^
IL-13	2.2 (0.6–57.6)	3.3 (0.7–51.0)	0.70^c^

*P* values according to ^a^Chi-square test, ^b^independent *t*-test, and ^c^Mann-Whitney *U* test.

^aa^Chi-square test comparing use and nonuse of immunomodulatory treatment.

^*^One missing value.

IQ: interquartiles; CIS20r: Checklist Individual Strength; IL: interleukin; TNF*α*: tumor necrosis factor *α*; IFN: interferon.

**Table 2:** Unadjusted and adjusted regression coefficients (*β*) of log⁡ 10 transformed cytokine levels with CIS20r fatigue score as outcome.

	Unadjusted *β* (se)	Adjusted *β* (se)	*P* value *β*_adj_	Explained variance *R* ^2^	Adjustment for confounding by
Pro-inflammatory cytokines					
IL-1*β*	−2.250 (7.777)	−16.407 (10.719)	0.138	0.163	Age, type of MS, duration of MS, EDSS, DMDs
IL-2	−1.345 (6.008)	−2.676 (6.996)	0.705	0.019	Type of MS, EDSS, DMDs
IL-6	14.551 (6.748)^*^	19.642 (7.036)^*^	0.009	0.211	Age
IL-8	17.396 (11.875)	17.396 (11.875)	0.152	0.061	—
IL-12p70	1.074 (4.012)	−2.292 (5.383)	0.674	0.070	Age, type of MS, EDSS, DMDs
IL-17	8.067 (6.003)	9.011 (6.282)	0.161	0.064	EDSS
TNF-*α*	6.656 (18.071)	4.950 (18.824)	0.794	0.005	Duration of MS
IFN-*γ*	3.294 (5.806)	3.749 (6.261)	0.554	0.061	Age, type of MS, DMDs
Anti-inflammatory cytokines					
IL-4	−11.081 (11.980)	−20.440 (15.070)	0.186	0.106	Age, duration of MS, EDSS
IL-5	−1.479 (4.556)	−4.594 (5.673)	0.425	0.092	Age, gender, type of MS, duration of MS, DMDs
IL-10	−2.225 (4.655)	−6.806 (5.995)	0.267	0.136	Age, gender, type of MS, duration of MS, EDSS, DMDs
IL-13	−2.804 (5.097)	−7.884 (6.467)	0.233	0.113	Age, type of MS, EDSS, DMDs

^*^Significant *β*.
